# Paternal age increases the risk for autism in an Iranian population sample

**DOI:** 10.1186/2040-2392-1-2

**Published:** 2010-02-22

**Authors:** Roksana Sasanfar, Stephen A Haddad, Ala Tolouei, Majid Ghadami, Dongmei Yu, Susan L Santangelo

**Affiliations:** 1 Department of Psychiatry, Harvard Medical School, Boston, MA, USA; 2 Psychiatric and Neurodevelopmental Genetics Unit, Center for Human Genetic Research, Massachusetts General Hospital, Boston, MA, USA; 3 Diagnosis and Prevention, Special Education Organization, Tehran, Iran; 4 Research Center, Ministry of Education, Tehran, Iran; 5 Department of Epidemiology, Harvard School of Public Health, Boston, MA, USA

## Abstract

**Background:**

Autism is a neurodevelopmental disorder which is known to have a strong genetic component and is most likely oligogenic. However, the necessary role of environmental factors has been well documented. Prior research suggests that parental characteristics, such as age and level of education, may be associated with a risk of autism. Parental age has been shown to be associated with many disorders, such as schizophrenia, childhood cancer and fetal death. However, results from studies of parental age and autism are inconsistent.

**Methods:**

In the present study, we investigated the association of autism with parental age in 179 autism cases and 1611 matched cohort children from Iran. Each case was matched with nine cohort controls on parental education, sex, order of birth, consanguineous marriage, urbanism and province of residence. The Cox regression model was used to carry out conditional logistic regression on the matched data.

**Results:**

There was a significant association between higher paternal age, but not maternal age, and an increasing risk of autism. An analysis of the combined effect of parental age and education also revealed that parents with higher education had an increased risk of having autistic children, with a dose-response effect of parental age.

**Conclusions:**

This study, which is the first epidemiological study of autism in Iran, provides evidence of the association of paternal age and risk of autism.

## Background

Autism spectrum disorders (ASD) are a group of heterogeneous and fairly devastating neurodevelopmental disorders that include autism, Rett and Asperger syndromes and pervasive developmental disorder-not otherwise specified (PDD-NOS). Although several recent retrospective studies of toddlers indicated the presence of abnormalities in social, communication and play behaviour as early as 14 months of age [1-6], autism often remains undiagnosed until the age of 3 years or later. Children with autism have deficits in reciprocal social interaction and communication and display repetitive and stereotyped behaviours [7]. The prevalence of autism, which has seemingly increased dramatically during the last decade [8] is estimated at one or two per 1000 for autistic disorder [9,10] and five or six per 1000 for ASD [11], although the most recent estimates from the Centers for Disease Control in the USA indicate that as many as 1/150 8-year-olds in the USA may be affected [12].

The causes of autism are still unclear, although results from twin and family studies provide evidence for a strong genetic contribution, with the probability of multiple genetic loci involved [13]. Less than complete concordance in monozygotic twins reveals the necessary role of non-genetic factors in the aetiology of autism. As the population prevalence of ASD has increased, results from research on fertility in the USA have demonstrated a parallel increase in the population mean age of both maternity and paternity [14]. This trend, coupled with findings that parental age is associated with some genetic disorders, makes parental age a likely candidate for epidemiological investigation of autism.

The first indication that paternal age might be associated with a genetic disorder was revealed in 1912, when Weinberg discovered that dominant achondroplasia occurred mainly in last-born children [15]. However, it was Penrose who first showed that the association was due to advanced paternal age, but not maternal age or the child's birth order [16]. Paternal age at the birth of offspring has been associated with an increasing risk for several congenital disorders [17], including cleft lip and palate [18], syndactyly [19], situs inversus [20], hydrocephalus [21], Apert syndrome [22] and craniosynostosis [23]. Some other disorders associated with paternal age are childhood cancer [24,25], miscarriage [26], fetal death [27] and some autoimmune disorders [28]. Paternal age also appears to increase risk for both schizophrenia [29-34] and decreased intellectual capacity in offspring [35,36]. Advanced maternal age is associated with increased risk for chromosomal abnormalities such as Down's syndrome [37], and has been associated with brain damage during pregnancy [38], dyslexia [39] and idiopathic mental retardation [40].

Results from studies of increasing paternal and maternal age and risk of autism are diverse [41-47]. Only a few studies have focused primarily on parental age [48-50] or adjusted for the other parent's age [46,48,50]. Results include reports that the age of both parents is associated with autism [51], that paternal, but not maternal age, is associated [48,50], that maternal but not paternal age matters [45] and that there is no association of autism with parental age [46].

The present study is a case-cohort study, designed to investigate the association between parental age and risk of ASD in an Iranian population sample. Case and cohort comparison individuals were all preschool children, aged 4-11 (cases) or 5-7 (cohort comparisons), who were identified in the course of the first annual nation-wide screening of all preschool children in Iran for ASD and other developmental disorders. To our knowledge, this is the first Iranian epidemiological study of ASD; retrospective data are available from nearly 900,000 children per year since 1995.

## Materials and methods

Data for the present study come from the Children's Health and Evaluation Project (CHEP), sponsored by the Special Education Organization (SEO) of Iran. The CHEP conducts multidisciplinary screening and diagnosis of all Iranian preschool children every year in order to identify children with disorders that may adversely affect their educational outcome. This screening is mandatory and children's placement in schools is based on the results of the screening and diagnosis process. In the areas which participated in the CHEP, the coverage rate is close to 100%. However, since many remote villages and tribes were not included, CHEP achieved about 90% coverage nationwide in 2005; this coverage rate has increased in subsequent years.

Since 1995, almost a million preschool children have been screened each year for vision and hearing impairments, cognitive disabilities and musculoskeletal problems. More recently, a subset of these children were also screened for autism spectrum disorders. Children with identified disabilities are referred to specialists for appropriate interventions and to special classes, as indicated. In addition to data on preschool children, the CHEP also collects data on the children's parents, such as age, education, province of residence, whether the residence is in a rural or urban location and consanguinity.

### Autism screening and diagnosis

In 2005, we began to screen a subset of the preschool children for ASD. Therefore, 200,000 of a cohort of 866,893 children, between the ages of 5-11, who applied to begin attending school were screened for autism as part of the CHEP programme. The number of children screened for ASD has increased in subsequent years. However, CHEP has not yet instituted nationwide screening for all preschool children, since it is not yet possible to provide educational support and interventional services in several remote cities and villages of the provinces. Hence, only the 200,000 preschool children who were resident of capital cities of the targeted provinces in 2005 were screened for autism spectrum disorders.

This screening identified 219 possible ASD cases, aged 5-11. CHEP was not designed for the estimation of prevalence and the aim of screening was the placement of children for educational purposes. However, the prevalence estimate for ASD from the 2005 screening is one per 913 preschool children.

Although the children who were screened for ASD resided only in the capital cities of each of the targeted provinces, the comparison cohort is comprised of all children residing in any part of the targeted provinces that gave rise to the cases and who participated in CHEP and screened negative for ASD and all other disabilities. Preschool children from other provinces that did not participate in autism screening were excluded from study. The age range of the remaining cohort children was subsequently restricted to 5-7 years in order to eliminate any potential false negatives (children with an ASD who screened negative) as many older children (8-11 years) applying to enter school for the first time had previously been screened and referred to special classes, or other interventions, due to intellectual/cognitive disabilities or actual, or suspected, developmental disorders. Parents of these children may have hoped to mainstream the affected children by delaying their enrollment and undergoing screening again when the children matured. Some of the older children had never been screened before due to their parents' decision to delay screening in the hope that the child's developmental disability might resolve and, thereby, avoid a referral to special classes. After restricting the remaining cohort age range, excluding children with missing data on any of the critical variables, and including as cases seven clinically referred children who were 4 years old, the final sample was comprised of 179 ASD cases (aged 4-11 years) and 549,354 comparison children (5-7 years old) from 17 provinces in Iran.

The autism screening instrument used was the *Social Communication Questionnaire *(SCQ) [52]; the *Diagnostic and Statistical Manual of Mental Disorders *(fourth edition-text revision (DSM-IV-TR) [53], was the primary diagnostic tool. The SCQ, a valid test with high sensitivity and acceptable specificity [54], was standardized for the Iranian population [55]. The SCQ was adapted for use in Tehran - the most diverse city and representative of the entire country - by screening 502 normally developing children as well as 65 with autism, 104 with mental retardation and 41 children with other psychiatric disorders. In order to assess the diagnostic validity, we carried out factor analysis, correlation between individual items and total SCQ score, and receiver operator curve analyses. For differentiating autism from typically developing children, a score of 15 on the SCQ, with a sensitivity of 84% and specificity of 90%, was considered to be the most appropriate level. Every child who scored higher than 15 on the SCQ during the annual CHEP preschool screening was referred to a diagnostic centre for evaluation by a psychiatric specialist, who used DSM-IV-TR criteria, as well as observation of the child's behaviour, to make a clinical diagnosis of autism or ASD.

All clinical diagnoses were confirmed by Autism Diagnostic Interview-revised (ADI-R) [56,56]. The ADI-R was adapted for use in Tehran using the same procedures as described for the adaption of the SCQ. Adaptation samples were comprised of 100 autistic children, 100 typically developing and 50 children with mental retardation, in the 4-14 years age range. The reliability of the diagnostic and current algorithms of the ADI-R was assessed by estimating Cronbach's alpha. Test-retest reliability was assessed by estimating intraclass correlation coefficients in all domains and sub-domains, as well as for most of the items in the diagnostic algorithm. Intraclass correlation coefficients are in the range of 0.88-1.0. We estimated: Cronbach's α = 0.70 for the social domain score; 0.94 for communication in verbal children; 0.97 for communication in non verbal children; and 0.87 for the repetitive behaviour domain score. The validity of the ADI-R was assessed by comparing mean scores of domains and sub-domains, in both diagnostic and current algorithms, between autistic and typically developing children and also between children with autism and those with mental retardation by analysis of variance.

All of the cases were enrolled for cytogenetic evaluation and will also be included in a genome-wide association scan in the near future. All the families gave their informed consent for their children to participate in these studies. The ethical aspects of this study were considered according to the Declaration of Helsinki and approved by Special Education organization of Iran.

### Data analysis

Parental age was treated as both a continuous and a categorical measure. Five categories of paternal age were created: 19-24, 25-29, 30-34, 35-39 and 40 years old or older. Maternal age categories were: 13-19, 20-24, 25-29, 30-34, and 35 years old and older. Paternal age 25-29 and maternal age 20-24 were chosen as the reference categories based on physiological considerations and the robust number of cases in those age ranges. Parental education was investigated in four categories, which were the same for both fathers and mothers: graduate degree (4 years of college and higher), high school and associate's degree (high school graduates plus up to 2 years of college), primary and middle school attendees and uneducated parents who never attended school. In addition, three categories of birth order (first, second and third born and higher), three dichotomous variables (sex, consanguinity, and urbanism), as well as province of residence, were examined. Univariate tests of statistical significance include two-sided student's *t*-tests for the continuous variables and chi-squared tests for categorical variables.

As there were marked differences in the distributions of variables between case and cohort comparison children, particularly for fathers' and mothers' age, birth order and fathers' and mothers' education, a matched analysis was done in order to achieve a greater balance within the levels of potentially confounding variables. The variables used for matching also yielded a greater than 15% change in the odds ratio (OR) for paternal age in the unmatched regression analysis and, thus, were considered possible confounders. Nine comparison children were matched to each case for fathers' and mothers' education, birth order, sex, consanguinity, urbanism and province. The Cox regression model [57] was used in order to carry out conditional logistic regression on the matched data. Analyses were done using both the Statistical Package for the Social Sciences v. 15.0 (SPSS) [58] and Statistical Analysis Software (SAS) v.9.1 (SAS) [59].

## Results

Cases included children with autistic disorder (68%), or Asperger's syndrome, and PDD-NOS combined (32%), based on a child psychiatrist's diagnosis verification.

More than half of the 179 ASD cases were first born, and the male to female ratio was 4:1. As shown in Table 1, children with ASD were significantly more likely than their cohort comparisons to be male, and to have parents who were more highly educated.

**Table 1:** Sample demographics.

	Children with autism spectrum disorder (*N *= 179)	Comparison cohort (*N *= 549,354)	***p*-value**
**Mean paternal age**	33.3	32.0	0.542
**Mean maternal age**	27.5	26.9	0.126
**Paternal education**			
College graduate + (%)	35.6	10.9	0.000
< High school (%)	34.6	61.3	
**Maternal education**			
College graduate + (%)	30.7	7.3	0.000
< High school (%)	35.4	64.4	
**Birth order**			
First born (%)	55.3	43.6	0.003
**Sex**			
Male (%)	79.9	51.6	0.000
**Consanguinity (%)**			
Related parents	32.4	30.4	0.573
**Urban/rural**			
Urban (%)	97	79	0.000

### Unmatched analyses

Results from the categorical analyses of paternal age showed a significant positive association between risk of autism and paternal age of 40 and older, compared to the reference category of 25-29, after adjusting paternal age for all other covariates (Table 2). The risk of having a child with autism was lower for parents with a lower level of education compared to the more highly educated parents in both the unadjusted and adjusted analyses. As expected, the risk of autism was inversely associated with birth order (risk decreases with increasing birth order). Compared with urban residence, the risk of autism decreased for those living in rural areas. Maternal age and consanguinity (measured as dichotomous variables) were not significant predictors of autism risk (Table 2). Compared to Tehran (the capital of Iran) residence in certain provinces was associated with increased risk.

**Table 2:** Results of univariate and adjusted unmatched analyses.

			Univariate analysis		Adjusted analysis	
**Variable**	**Cases**	**Cohort Comparisons**	***p*-value**	**Odds ratio (95% confidence interval)**	***p*-value**	**Odds ratio (95% confidence interval) 1**

**Fathers' age**						
19-24	**10**	**53497**	.267	0.679 (0.343-1.344)	0.796	0.912 (0.453-1837)
25-29	**47**	**170796**	Ref.			
30-34	**57**	**167541**	.282	1.236 (0.840-1.819)	0.832	1.046 (0.690-1.586)
35-39	**38**	**95519**	.091	1.446 (0.943-2.217)	0.167	1.435 (0.860-2.395)
=> 40	**27**	**62001**	.057	1.583 (0.986-2.541)	**0.023**	2.026 (1.100-3.728)
**Mothers' age**						
13-19	**7**	**43320**	.189	0.587 (0.265-1.299)	0.442	0.727 (0.324-1.636)
20-24	**47**	**170732**	Ref.			
25-29	**63**	**167423**	.105	1.367 (0.937-1.994)	0.528	1.143 (0.775-1.731)
30-34	**43**	**108886**	.087	1.435 (0.949-2.170)	0.699	1.108 (0.659-1861)
=> 35	**19**	**58993**	.564	1.170 (0.687-1.993)	0.636	0.846 (0.422-1.694)
Fathers' education*						
Graduate	**64**	**69656**	Ref.			
High school	**53**	**153123**	**.000**	**. 323 (.224-. 464)**	**0.009**	**0.578 (0.383-0.873)**
Primary/middle school	**48**	**288446**	**.000**	**.155 (.107- .226)**	**0.004**	**0.462 (0.273-0782)**
No education	**14**	**48129**	**.000**	**.271 (.152- .483)**	0.984	0.992 (0.430-2.289)
* **Mothers' education**						
Graduate	**55**	**40106**	Ref.			
High school	**57**	**155404**	**.000**	**.267 (.185 - .387)**	**0**	**0.438 (0.284-0.674)**
Primary/middle school	**51**	**281892**	**.000**	**.132 (.090- .193)**	**0.001**	**0.390 (0.222-0685)**
No education	**16**	**71952**	**.000**	**.162 (.093- .283)**	0.101	0.488 (0.207-1.152)
**Birth order**						
First born	**99**	**239361**	Ref.			
Second	**48**	**160025**	0.068	.725 (.514 - 1.024)	**0.05**	**0.681 (0.464-1.000)**
Third & higher	**32**	**149968**	**0.001**	**.516 (.346 - .769)**	**0.016**	**0.515 (0.300-0.884)**
**Sex**						
Female	**36**	**266124**	Ref.			
Male	**143**	**283230**	**.000**	**3.732 (2.590-5.379)**	**0**	**3.358 (2.607-5.417)**
**Consanguinity**†						
not related	**121**	**382301**	0.562	1.097 (0.802-1.500)	**0.003**	**1.639 (1.182-2.274)**
related	**58**	**167053**	Ref.			
**Urban/Rural**						
Rural	**4**	**115499**	.000	.086 (0.032-.0231)	**0.001**	**0.163 (0.059-0.454)**
Urban	**175**	**433855**	Ref.			

* Graduate indicates college graduate up to and including graduate degrees. High school indicates high school graduate up to associate's degree (2 years college); primary/middle school indicates any education up to but not including high school.

†Consanguinity (scored dichotomously) indicates that the parents are closely related, as either first, second or third cousins.

**Figure 1:**
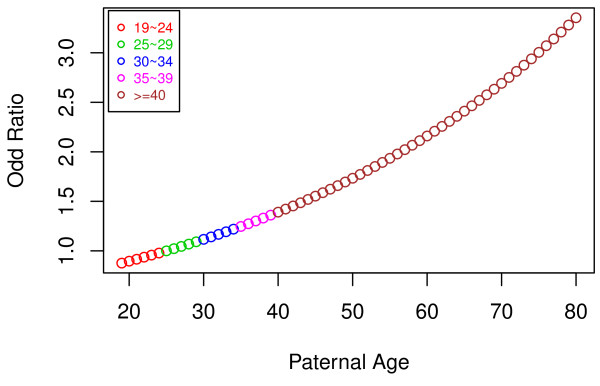
**Paternal age modelled as a continuous variable**. Point estimate of odds ratio.

Paternal age was also modelled as a continuous variable in the unmatched data. In these analyses, autism risk increased by 29% for every 10 year increase in age for fathers, adjusting for all other potential confounders [1.027 (1.006-1.048) for each year of age]. In the adjusted analyses of paternal age treated as a categorical variable, paternal age of 40 and older was significantly associated with autism [OR = 2.03 (1.10-3.73)]. Figure 1 displays this increased risk graphically showing the OR of having an autistic child at various paternal ages compared with the risk associated with paternal age of 25.

### Matched analysis results

**Figure 2:**
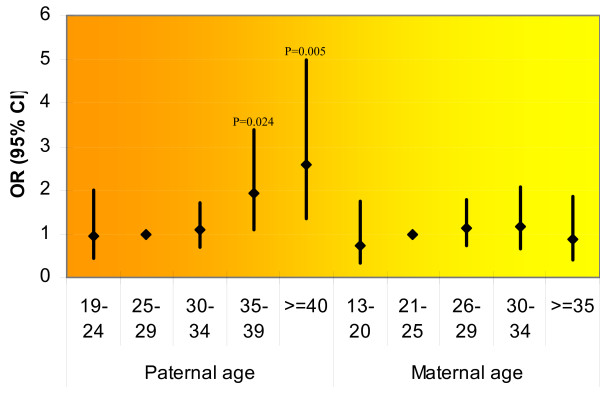
**Matched analysis results**. Point estimate of odds ratio; bar indicates 95% confidence limits around point estimate; *p*-value < 0.05.

There was a significant effect of paternal age on the risk of autism in the matched analysis. Fathers aged 35-39 had a nearly twofold (1.92 [1.09-3.37]) greater risk and a higher than twofold (2.58 [1.34-4.97]) greater risk for those aged 40 and older at the birth of the child, than those in the reference category (25-29 years) (Figure 2). This association was independent of maternal age. No significant association between maternal age and risk of autism was found (*p *= 0.77). Paternal and maternal age were significantly correlated (*r *= 0.615, *p *< 0.001).

### Combined effect of parental age and education

As we had seen an increase in risk with both paternal age and parental education in the unmatched analyses (Table 2), we decided to conduct a joint analysis of parental age and education. Paternal age > 35 and maternal age > 30 were designated as the potential risk categories (older parents). A new variable with three categories was created using the unmatched data: both parents younger (women younger than 30 and men younger than 35); only one parent older (either a mother or father older than the reference age category of 30 for women and 35 for men); and both parents older (both parents in a risk age category). The reference group for this new variable is 'both parents younger'. A similar variable was created for parental education level, with five categories, depicting the highest combined level of education of the two parents: (1) both parents college graduates or better (CG); (2) father a college graduate and mother with only high school or primary school (HS/PS); (3) mother a CG and father with HS/PS; (4) either both parents HS or one with HS and the other PS or uneducated; and (5) both parents uneducated. Finally, the new age and education variables were combined into 15 categories.

**Figure 3:**
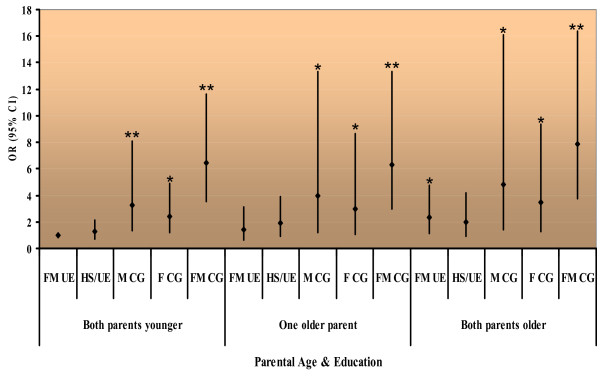
**Combined parental age and education**. Point estimate of odds ratio; bar indicates 95% confidence limits around point estimate; * *p*-value < 0.05; ** *p*-value < 0.005. FM UE, Father and mother uneducated; HS/UE, both parents high school, or one high school and the other uneducated; F CG: father has 4 or more years college and mother, high school or uneducated; M CG, mother has 4 or more years college and father, high school or uneducated; FM CG, both parents have 4 or more years of college.

Compared to younger, uneducated parents, older parents with college degrees or better had a six to eightfold higher risk of having a child with ASD (95% CI: 3.77- 16.36); the effect of maternal education was stronger than the effect of paternal education (Figure 3). Within each parental combined age category (both parents younger, one parent older, both parents older) there was a more or less linear trend for an increasing effect of education. This effect remained after adjusting for birth order, sex, consanguinity, urbanism and province of residence, and was significant for all but the lowest education categories, except when both parents were in the older range. In the case of two older parents, even the lowest education category (both parents uneducated) carried a significantly increased risk for autism. In fact, for two older parents, all but the second education category (where both parents are high school graduates or one is a high school graduate and the other is uneducated) conferred significant risk for producing a child with autism.

## Discussion

In this population-based study of data from the large CHEP in Iran we found that risk of ASD was associated with paternal but not maternal age. Although CHEP was not designed as a prevalence study, the first CHEP conducted in 2005 found a prevalence for ASD of one per 913, which is lower than in the USA and Europe. This is, in part, because the screening relied on the SCQ parent questionnaire. For all other developmental disabilities CHEP screening has always been conducted directly with the children and has not previously relied exclusively on parent reports. We suspect that screening via parent report resulted in an artificially low positive screen for ASD prevalence.

As noted above, the results from different investigations of paternal age and risk of autism are inconsistent. Our result is inconsistent with epidemiological studies that found that both paternal and maternal age, or only maternal age but not paternal age, were associated with increased risk for autism. This inconsistency may be explained by differences in study design, the specific variables investigated and adjusted for, as well as sample characteristics. For example, Glasson *et al*. [45] found an association of advanced maternal age and increased risk for autism. As this result was obtained as part of their epidemiological study on obstetric factors, no data were available on paternal age. Consequently, maternal age was not adjusted for paternal age in this study.

However, Croen *et al*. [49], in a historical birth cohort study in northern California, reported that the risk of autism increased significantly with each 10-year increase in both maternal and paternal age, although the effect was stronger for maternal age. Diagnostic subgroup analyses showed a slightly higher risk with increasing maternal age in the Asperger's syndrome and PDD-NOS subgroups compared to the autistic disorder group and showed an increased risk with paternal age only for autistic disorder but not Asperger's syndrome or PDD-NOS [49].

The unmatched analysis of our data showed that the risk of having a child with ASD increases 29% for every 10 years of increase in paternal age, after adjusting for all other covariates. However, we found no significant association with maternal age in either the unmatched, adjusted analyses or the matched analyses. Although we were unable to conduct analyses of the effect of paternal and maternal age within the diagnostic subgroups of ASD, due to an inadequate number of cases in each of the subgroups, this inconsistency for the effect of maternal age may be due to a difference in the proportion of diagnostic subgroup cases in the sample studied by Croen *et al*. and the present study sample. Therefore, a higher proportion of autism cases, compared to Asperger's syndrome and PDD-NOS cases, in our sample may account for our finding of increased risk with paternal, but not maternal, age. On the other hand, a higher proportion of Asperger's syndrome and PDD-NOS cases may account for the results from other studies that have reported either both paternal and maternal age or only maternal age associated with increased risk of autism. Along these lines, it is of interest that a recent study in Japan found that advanced paternal age but not maternal age was associated with increased risk of high-functioning autism without intellectual disability [29]. Thus, the variable distribution of diagnostic subgroups composing a sample of ASD cases, as well as the distribution of intellectual ability among the cases, may be one reason for the inconsistency of results in studies of the effect of paternal and maternal age on ASD. However, it is by no means clear how or why either paternal or maternal age should be associated with any one diagnostic subgroup in particular.

It is clear that, in addition to genetic risk, environmental factors must also be important in the aetiology of ASD. Of course, specific environmental risk factors may be associated with other environmental and/or genetic risk factors which, although they may be unknown or unmeasured, may nonetheless serve as modifiers or confounders. As mentioned earlier, matched analysis has the advantage of being able to control for potential confounders that are difficult or impossible to measure directly and which, therefore, cannot be adjusted for in an unmatched analysis.

An example of a potential confounder that is possibly more important in Middle Eastern populations is consanguinity. The prevalence of consanguineous marriage (between cousins) in Iran is about 20%-30% and is associated with a higher rate of child marriage (marriage before age 18) and a lower socio-economic status. Child marriage is itself associated with: a reduced opportunity to provide education for all family members, including parents; lower parental education level; a higher number of offspring; and a generally lower standard of living. In addition, child marriage (3% prevalence for men and 20% for women in Iran) [60,61] results in lost educational opportunities, interrupted bodily development (due to early pregnancy), less awareness of the importance of self-care and pre-natal care, generally poorer health, a greater compliance with authority figures and a higher exposure to abuse for women. All of these can have an effect on the outcome of pregnancy [62-64]. In many cases, child marriage results in a greater age disparity between the two parents, as, for instance, when a 12-year-old girl is married to a 22-year-old man. Each of these variables may have its own confounding or modifying effect on parental age and autism. They may also interact with each other and with other environmental and/or genetic risk factors that are unknown or unmeasured. While most published studies controlled for potential measured confounders, few conducted matched analyses which, because they are better able to control for unknown or unmeasured potential confounders, are more precise. As we were concerned about the effects of known but unmeasured variables, particularly socio-cultural factors, we conducted a matched analysis, matching each case with nine cohort comparison children on parental education, birth order, sex, consanguinity, urbanism and province. Thus, the present study is one of the few to conduct a matched analysis examining the effect of parental age on autism risk.

Larsson and colleagues [46], in a matched study on prenatal risk factors and autism, found no association of parental age and risk of autism, after matching on all other prenatal factors, socioeconomic status and parental psychiatric history. They matched each case with 25 controls for sex, year of birth and age from a cohort of all children born in Denmark since 1973 who had received a diagnosis of autism not later than the end of December 1999. However, it is notable that when the Larson study matched for only prenatal risk factors but not parental psychiatric history, they found paternal age to be associated with a risk of autism. Thus, parental psychiatric status may be an independent risk factor or confounder, although we were unable to control for this in our study.

Many negative outcomes of pregnancy have been attributed to the age-associated increased risk of spontaneous mutation in male sperm [16,65]. In addition to the many studies that have confirmed the association of advanced paternal age and de novo germ-line mutations, a recent analysis of spermatozoa from men of different ages showed a small increase in mutant sperm with paternal age [61] which, nevertheless, is likely to have a significant disease burden. In addition, another study using fluorescence *in situ *hybridization analysis of spermatozoa identified several potential paternal risk factors for increasing chromosomal abnormalities, including age, drug use, lifestyle and occupational exposure [66]. However, most epidemiological studies of parental age and autism lack data on such specific potential confounders or modifiers. Finally, another alternative explanation is the potential for the accumulation of toxic chemical exposures in older fathers and/or the potential for inherited epimutation. However, there is very little information about either of these potential causes in Iran.

We also investigated the joint effect of parental age and education. Combining parental age with parental education, we found that, regardless of parental age, a higher risk of having a child with autism was found among couples when the mother had a college or graduate degree. However, the highest risk of having a child with autism was found in couples who were both college graduates, with a dose-response effect of parental age (Figure 2). The increased risk from a combination of advanced parental age and greater level of education may be due to greater educational support seeking behaviours in more educated parents. That is, the more highly educated parents may have been more motivated to participate in this first nationwide screening and diagnosis for autism, whereas those with less education may have been more likely to keep their affected children out of the education system and, thus, did not participate in the screening project. In fact, when we divided our cases into two age categories, those aged 4-7 and those aged 8-11, and compared their parents' education, we found that older cases were significantly more likely than younger cases to have fathers with higher education [χ^2^_3 _= 8.718 (*p *= 0.033)], but not more highly educated mothers [χ^2^_3 _= 1.915 (*p *= 0.59)].

There were, of course, some limitations to our study design. The main limitation of our study was the small number of cases, which made it impossible for us to conduct diagnostic subgroup analyses. Other limitations include the lack of information on parents' autistic traits, and on environmental risk factors for parents and children.

## Conclusions

Our study indicates a clear and significant association of advanced paternal age and the risk of ASD in an Iranian population sample. This finding was observed in both an unmatched data analysis, after adjusting for several potential confounders, as well as a matched analysis, with nine matching factors. *De novo *germ line mutations may be responsible, at least in part, for this association. Socio-cultural characteristics of the Iranian population, such as a large age difference between the parents of a child, child marriage and consanguineous marriage may, in part, account for the inconsistency between the results of our study and some others. Future studies should attempt to conduct subgroup analyses, particularly for subgroup diagnosis and intellectual ability. These results await replication in a larger case group from the same population. In such a replication sample, it would be ideal to have information on broader autism phenotype traits in the parents, as well as environmental risk exposures for both parents and children.

## Abbreviations

ADI-R: Autism Diagnostic Interview - revised; ASD: autism spectrum disorder; CG: college graduate; CHEP: Children's Health and Evaluation Project; DSM-IV-TR: Diagnostic and Statistical Manual of Mental Disorder, 4th edition, text revision; HS: high school; PDD-NOS: pervasive developmental disorder not otherwise specified; PS: primary school; SCQ: Social Communication Questionnaire; SEO: Special Education Organization.

## Competing interests

The authors declare that they have no competing interests.

## Authors' contributions

RS designed the study, acquired the data, performed most of the data analyses and drafted the manuscript. SH carried out data cleaning and data analysis. AT and MG helped with data acquisition. DY helped with study design and the matched data analysis. SLS contributed to the study design, analysis and interpretation and substantially revised and edited the manuscript.

## Authors' information

RS is an Iranian physician, currently working in the USA. She was instrumental in initiating nationwide screening for ASD in Iran, when she worked with the Special Education Organization of Iran. SLS is a genetic epidemiologist with over 16 years of experience in autism research.
